# The Genetic Basis and Nutritional Benefits of Pigmented Rice Grain

**DOI:** 10.3389/fgene.2020.00229

**Published:** 2020-03-13

**Authors:** Edwige Gaby Nkouaya Mbanjo, Tobias Kretzschmar, Huw Jones, Nelzo Ereful, Christopher Blanchard, Lesley Ann Boyd, Nese Sreenivasulu

**Affiliations:** ^1^International Rice Research Institute, Los Baños, Philippines; ^2^International Institute for Tropical Agriculture, Ibadan, Oyo, Nigeria; ^3^Southern Cross Plant Science, Southern Cross University, Lismore, NSW, Australia; ^4^National Institute of Agricultural Botany, Cambridge, United Kingdom; ^5^School of Biomedical Sciences, Charles Sturt University, Wagga Wagga, NSW, Australia

**Keywords:** pigmented rice grain, nutrition, flavonoids, metabolites, genetics

## Abstract

Improving the nutritional quality of rice grains through modulation of bioactive compounds and micronutrients represents an efficient means of addressing nutritional security in societies which depend heavily on rice as a staple food. White rice makes a major contribution to the calorific intake of Asian and African populations, but its nutritional quality is poor compared to that of pigmented (black, purple, red orange, or brown) variants. The compounds responsible for these color variations are the flavonoids anthocyanin and proanthocyanidin, which are known to have nutritional value. The rapid progress made in the technologies underlying genome sequencing, the analysis of gene expression and the acquisition of global ‘omics data, genetics of grain pigmentation has created novel opportunities for applying molecular breeding to improve the nutritional value and productivity of pigmented rice. This review provides an update on the nutritional value and health benefits of pigmented rice grain, taking advantage of both indigenous and modern knowledge, while also describing the current approaches taken to deciphering the genetic basis of pigmentation.

## Introduction

Rice is a staple food for over half of the world’s population (World Rice Production, 2019). Meeting the demand of future rice supply for the growing population, which has been predicted to reach 9.7 billion by 2050^1^, is central for ensuring food and nutritional security. In addition to its critical importance to Asian populations as a source of food, rice also features in a range of social, cultural, economic, and religious activities (Ahuja et al., 2007; Hedge et al., 2013; Sathya, 2013). In sub-Saharan Africa the consumption of rice is projected to grow from its current level of 27–28 Mt per year to around 36 Mt by the end of 2026 (Terungwa and Yuguda, 2014; Nigatu et al., 2017), replacing some of the current demand for cassava, yam, maize, millet, and sorghum.

Most of the nutrients found in rice grain accumulate in the outer aleurone layer and embryo, the endosperm being composed primarily of starch. The process of dehulling and milling discards most micronutrients, fatty acids, anti-oxidants, and fiber. As a result, diets over-reliant on white rice risk deficiencies for several nutritional factors (Verma and Shukla, 2011; Sharma et al., 2013; Saneei et al., 2016; Sarma et al., 2018). The focus of rice breeding has long been concentrated on improving the crop’s productivity, although some emphasis has been given to improving the size, shape, and amylose content of the grain (Breseghello, 2013; Rao et al., 2014). The nutritional quality of the grain produced by certain traditional landraces has been shown to be higher than that of the grain produced by conventional, modern rice varieties, largely due to their more effective accumulation of bioactive compounds (Bhat and Riar, 2015; Berni et al., 2018). A growing consumer interest in health-promoting food products is generating a substantial market for more nutritionally valuable rice, creating health benefits for the large number of people for whom rice is a staple, while simultaneously generating economic benefits for the producers (Terungwa and Yuguda, 2014). As a result, the focus of a number of major rice research programs is turning to the issue of nutritional quality, encompassing an improved micronutrient and anti-oxidant content, along with a reduction in the grains’ glycemic index.

This review explores the nutritional and health attributes of pigmented rice grain, based on both indigenous knowledge and current research, and discusses the potential of pigmented rice grain to address nutritional food security. In addition, it explores the genetic basis of grain pigmentation, and suggests the potential contribution which ‘omics technologies can make to address the challenge of the double burden of malnutrition.

## Indigenous Knowledge, Complemented With Corroborated Scientific Evidence, Informs on the Potential of Pigmented Rice Grain to Improve Nutrition and Health

**FIGURE 1:**
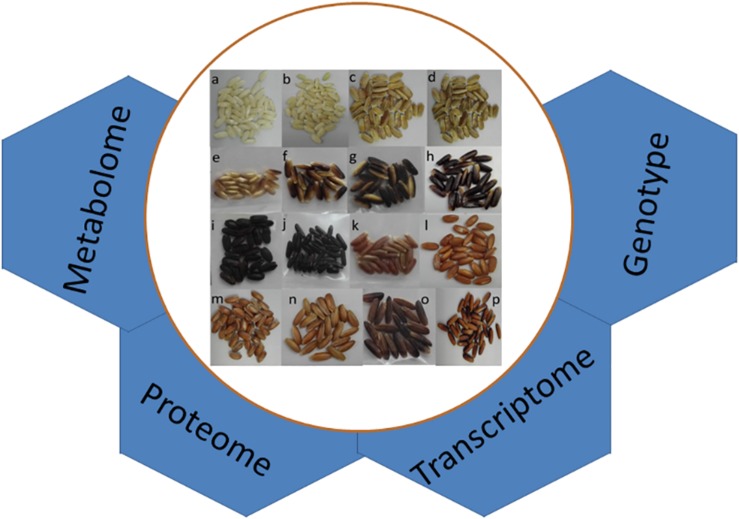
Genetic variation for grain pigmentation in rice. Grains featuring **(a,b)** white, **(c,d)** brown, **(e–h)** purple, **(i,j)** dark purple or black, **(k–n)** red, and **(o,p)** mixed colored pericarp. The application of various genomic approaches to understand the genetic pathway of grain pigmentation is outlined.

Indigenous diets have developed to meet the needs of local communities over a long period of time, and the knowledge associated with these should be viewed as a resource to inform the discussion concerning the place of rice in the modern diet (Berni et al., 2018; Khatri, 2018). The value of such landraces in the context of both human nutrition and health (Rahman et al., 2006; Chunta et al., 2014) can be exemplified by the proven advantages of consuming pigmented grain (Figure 1; Rahman et al., 2006; Umadevi et al., 2012; Sathya, 2013). In particular, pigmented rice has been associated with anti-inflammatory and diuretic activity (Umadevi et al., 2012). Based on native indigenous knowledge, it has also been recommended for the treatment of diarrhea, vomiting, fever, hemorrhaging, chest pain, wounds, burns, and gastrointestinal problems, as well as addressing various liver and kidney disorders (Hedge et al., 2013; Sathya, 2013). Certain pigmented rice varieties are still used to treat skin diseases, blood pressure, fever, paralysis, rheumatism, and leucorrhea, and even as the basis of a general health tonic (Ahuja et al., 2007). In the Philippines, “tiki tiki,” derived from rice bran, has been used to cure thiamine deficiency (Umadevi et al., 2012). In India, the grain of pigmented rice landraces is offered to lactating mothers, and is used to both treat jaundice and cure paralysis. The rice variety “Laicha” was given its name because of its ability to prevent an eponymous skin disease (Das and Oudhia, 2000). For more than 2,000 years, grain of the South Indian landrace “Kavuni” has been reported to exhibit anti-oxidant, anti-arthritic, and anti-diabetic properties, and has been used to cure gastritis and peptic ulcers, as well as to enhance blood circulation (Valarmathi et al., 2014; Hemamalini et al., 2018).

A number of scientifically based studies have provided evidence to support the hypothesis that pigmented rice grain possesses anti-oxidant, anti-diabetic, anti-hyperlipidemic, and anti-cancer activity (Baek et al., 2015; Boue et al., 2016), which is reviewed below.

### Anti-oxidant Activity

Dietary anti-oxidants represent an effective means of combating the accumulation of harmful reactive oxygen species and of balancing the redox status of the body (Krishnanunni et al., 2014). Analysis of extracts made from pigmented rice grain has shown that the phenolic compounds tocopherol and anthocyanin are efficient neutralizers of reactive oxygen species (Zhang et al., 2015; Ghasemzadeh et al., 2018), while animal tests have proven that these compounds are bioavailable (Tantipaiboonwong et al., 2017). Several studies have shown that the elevated anti-oxidation activity exhibited by pigmented rice grains (most markedly by black rice) can be used to mitigate the inflammatory response (Chakuton et al., 2012; Petroni et al., 2017).

### Anti-diabetic Activity

The grain of some traditional pigmented rice varieties have proven to be effective in supporting glucose homeostasis, and are thus useful for the management of diabetes mellitus (Hemamalini et al., 2018). Unlike white rice grain consumption, which raises blood glucose levels, consuming pigmented grain can reduce blood glucose levels. Extracts of pigmented rice grain and bran have been shown to effectively inhibit the activity of endogenous α-amylase and α-glucosidase, thereby inhibiting the conversion of starch to glucose in the small intestine, which acts as a source of resistant starch to be utilized by gut microbiota in the colon (Boue et al., 2016; Chiou et al., 2018). While extracts made from both red and purple grain have been reported to inhibit α-glucosidase activity, only the former was effective in also inhibiting α-amylase activity (Valarmathi et al., 2014; Boue et al., 2016). The anthocyanins found in the whole grain of black rice acted as a potent inhibitor of β-glucosidase, thus delaying the absorption of carbohydrates (Chandramouli et al., 2018). Extracts of black rice bran have also been shown to induce the repair and regeneration of pancreatic beta cells (Wahyuni et al., 2016). Overall, the anti-diabetic effects of pigmented rice seem to arise from a synergistic effect of anthocyanin, proanthocyanidin, vitamin E, γ-oryzanol, and various flavonoids (Tantipaiboonwong et al., 2017). Black rice extracts reduced blood glucose levels more quickly than did extracts from red rice, a difference which was attributed to the presence of cyanidin 3-glucoside, a compound which activates insulin sensitivity, glucose uptake, and adiponectin secretion (Matsukawa et al., 2016; Tantipaiboonwong et al., 2017). However, many of the black rice are low in its amylose content and upon milling most of the anthocyanins accumulated in aleurone will be lost, thus not necessarily would possess low GI property when consumed in the form of milled rice (Kang et al., 2011).

### Anti-cancer Activity

A considerable body of evidence suggests that consumption of pigmented rice has a protective effect against certain cancers. Ghasemzadeh et al. (2018) demonstrated that extracts of both black and red rice inhibit the proliferation of breast cancer cells. The phenolic acids, flavonoids, anthocyanins, and phytic acid present in extracts of purple rice bran have been shown to act as anti-mutagens and potential suppressors of cancer. It has been proposed that these phytochemicals act by either blocking the carcinogenetic cytochromes P450 CYP1A1 and CYP1B1 and/or by effectively scavenging free radicals (Insuan et al., 2017). Bioactive compounds of pigmented grains can reduce the viability of cancer cells and even induce their apoptosis. The mechanistic basis of this effect has been found to be variety-dependent, reflecting differences in the spectrum of bioactive compounds present in each rice variety (Baek et al., 2015). The high anthocyanin content of purple rice has been associated with an inhibitory effect on the growth of human hepatocellular carcinoma cells (Banjerdpongchai et al., 2013), while extracts of purple rice bran were able to block the first stage of aflatoxin B1-initiated hepatocarcinogenesis by inhibiting key metabolic activating enzymes (Suwannakul et al., 2015). Extracts of red rice have been shown to limit the invasiveness of cancer cells in a dose-dependent manner (Pintha et al., 2014). The phytosterols 24-methylenecycloartanol, β-sitosterol, gramisterol, campesterol, stigmasterol, cycloeucalenol, 24-methylene-ergosta-5-en-3β-ol, and 24-methylene-ergosta-7-en-3β-ol, all of which are present in extracts of black rice bran, have also been reported to be effective as agents restricting the proliferation of murine leukemic cells (Suttiarporn et al., 2015). Consequently, one of the long-term strategies proposed by Luo et al. (2014) to prevent breast cancer metastasis relies on the inclusion of pigmented rice in the human diet.

## The Biochemical Properties of Pigmented Rice Grain

### Phytosterols, Carotenoids, Vitamins, and Micronutrients in Pigmented Rice Grain

#### Phytosterols

Rice grains contain a wide range of secondary metabolites (Table 1). Pigmented grain appears to accumulate a higher level of γ-oryzanol than does non-pigmented grain (Chakuton et al., 2012). The grain accumulates the active anti-oxidant γ-oryzanol, which comprises a mixture of several phytosteryl ferulates (Chakuton et al., 2012), in particular 24-methylenecycloartanyl ferulate, cycloartenyl ferulate, campesteryl ferulate, and β-sitosteryl ferulate (Zubair et al., 2012; Pereira-Caro et al., 2013). The most important nutritional benefit of the phytosterols is their ability to both inhibit the absorption of cholesterol and to control the blood’s content of undesirable lipoproteins (Jesch and Carr, 2017). The predominant phystosterols detected in commercial rice varieties are β-sitosterol, followed by campesterol, Δ^5^-avenasterol, and stigmasterol (Zubair et al., 2012). The bran of the black rice variety “Riceberry” also harbors three additional sterols, namely 24-methylene-ergosta-5-en-3β-ol, 24-methylene-ergosta-7-en-3β-ol, and fucosterol (Suttiarporn et al., 2015).

**TABLE 1:** Bioactive and nutritional compounds identified in pigmented rice.

Compound	PubChem CID	Compound class	Figure 2^a^	References
Cyanine 3-glucoside	197081	Anthocyanin	✓	Pereira-Caro et al., 2013;
Peonidin-3-glucoside	443654	Anthocyanin	✓	Zhang et al., 2015;
Cyanidin	128861	Anthocyanin		Tantipaiboonwong et al., 2017;
Cyanidin-3,5-diglucoside	44256718	Anthocyanin	✓	Shao et al., 2018
Cyanidin-3-*O*-(6”-*O*-*p*-coumaroyl)glucoside		Anthocyanin	✓	
Pelargonidin-3-*O*-glucoside	443648	Anthocyanin		
Peonidin-3-*O*-(6”-*O*-*p*-coumaroyl)glucoside		Anthocyanin	✓	
Cyanidin-3-*O*-arabidoside		Anthocyanin	✓	

Flavone	10680	Flavone		
Luteolin-6/8-*C*-pentoside-6/8-*C*-hexoside (2 isomers)		Flavone		Pereira-Caro et al., 2013;
Apigenin-6/8-*C*-pentoside-8/6-*C*-hexoside (three isomers)		Flavone glycoside		Kim et al., 2014;
Apigenin-6-*C*-glucosyl-8-*C*-arabinoside		Flavone		Ghasemzadeh et al., 2018;
Tricin-*O*-rhamnoside-*O*-hexoside		Flavone		Poulev et al., 2019
Tricin	5281702	Flavone	✓	
Chrysoeriol	5280666	Flavone	✓	
Luteolin	5280445	Flavone	✓	
Apigenin	5280443	Flavone	✓	

Caffeic acid	689043	Hydrocinnamic acid	✓	Gunaratne et al., 2013;
p-Coumaric acid	637542	Hydrocinnamic acid	✓	Zhang et al., 2015;
Ferulic acid	445858	Hydrocinnamic acid	✓	Irakli et al., 2016;
Sinapic acid	637775	Hydrocinnamic acid	✓	Tantipaiboonwong et al., 2017;
Isoferulic acid	736186	Hydrocinnamic acid		Chiou et al., 2018;
Chlorogenic acid	1794427	Hydrocinnamic acid		Ghasemzadeh et al., 2018; Shao et al., 2018

2,5-Dihydroxybenzoic acid	3469	Hydroxybenzoic acid	✓	Kim et al., 2014;
*p*-Hydroxybenzoic acid	135	Hydroxybenzoic acid	✓	Valarmathi et al., 2014;
Gallic acid	370	Hydroxybenzoic acid	✓	Suwannakul et al., 2015;
Vanillic acid	8468	Hydroxybenzoic acid	✓	Huang and Lai, 2016;
Syringic acid	10742	Hydroxybenzoic acid	✓	Irakli et al., 2016;
Protocatechuic acid	72	Hydroxybenzoic acid	✓	Tantipaiboonwong et al., 2017;
Salicylic acid	338	Hydroxybenzoic acid	✓	Ghasemzadeh et al., 2018;
β-Resorcylic acid	1491	Hydroxybenzoic acid		Shao et al., 2018

Protocatechualdehyde	8768	Phenolic aldehyde		Huang and Lai, 2016
8-5′-Coupled diferulic acid		Phenolic dehydrodimer		Zhang et al., 2015
5-5′-Coupled diferulic acid		Phenolic dehydrodimer		
8-8′-Coupled diferulic acid benzofuran form		Phenolic dehydrodimer		

Proanthocyanidin dimer		Proanthocyanin	✓	Gunaratne et al., 2013
Proanthocyanidin trimer		Proanthocyanin	✓	

Catechin	73160	Flavanonol	✓	Tantipaiboonwong et al., 2017;
Epicatechin	72276	Flavanonol	✓	Ghasemzadeh et al., 2018

Quercetin	5280343	Flavonol		Pereira-Caro et al., 2013;
Quercetin-3-*O*-glucoside		Flavonol		Valarmathi et al., 2014;
Quercetin-3-*O*-rutinoside		Flavonol		Chiou et al., 2018;
Isorhamnetin-3-*O*-glucoside	5318645	Flavonol		Ghasemzadeh et al., 2018;
Myricetin	5281672	Flavonol		Poulev et al., 2019
Rutin	5280805	Flavonol		
Kaempferol	5280863	Flavonol		
Kaempferide	5281666	Flavonol		
Naringenin	932	Flavanone	✓	Chiou et al., 2018
Cycloartenol ferulate	134695320	γ-Oryzanol		Chakuton et al., 2012;
24-Methylenecycloartenol ferulate	9920169	γ-Oryzanol		Gunaratne et al., 2013;
Campesteryl ferulate	15056832	γ-Oryzanol		Pereira-Caro et al., 2013
β-Sitosteryl ferulate	9938436	γ-Oryzanol		
Δ7-Campesteryl ferulate		γ-Oryzanol		
Campestanyl ferulate	13786591	γ-Oryzanol		
Sitostanyl ferulate	11227138	γ-Oryzanol		

Phytic acid	890	Phytic acid		Chakuton et al., 2012; Insuan et al., 2017

Tocotrienols (α-, β-, γ-, δ-forms)	9929901	Vitamin E		Zubair et al., 2012;
Tocopherols (α-, β-, γ-, δ-forms)	14986	Vitamin E		Gunaratne et al., 2013; Irakli et al., 2016

Riboflavin	493570	Vitamin B2		Valarmathi et al., 2014

Nicotinic acid	938	Vitamin B3		Kim et al., 2014

Lutein	5281243	Carotenoid		Pereira-Caro et al., 2013;
Zeaxanthin	5280899	Carotenoid		Valarmathi et al., 2014;
β-Carotene	5280489	Carotenoid		Irakli et al., 2016;
Lycopene	446925	Carotenoid		Melini and Acquistucci, 2017
β-Carotene-4,4′-dione		Carotenoid		
all-trans-3,3’,4,4’-Tetrahydrospirilloxanthin	5366411	Carotenoid		
10′-Apo-β-carotenoic acid		Carotenoid		
24-Methylene-ergosta-5-en-3β-ol		Phytosterol		Suttiarporn et al., 2015
24-Methylene-ergosta-7-en-3β-ol		Phytosterol		
Fucosterol	5281326	Phytosterol		
Gramisterol	5283640	Phytosterol		
Campesterol	173183	Phytosterol		
Stigmasterol	5280794	Phytosterol		
β-Sitosterol	222284	Phytosterol		

Cycloeucalenol	101690	Triterpenoid		Suttiarporn et al., 2015
Lupenone	92158	Triterpenoid		
Lupeol	259846	Triterpenoid		
24-Methylenecycloartanol	94204	Triterpenoid		
LysoPC 14:0	460604	Phospholipid		Kim et al., 2014
LysoPC 18:2	11005824	Phospholipid		
LysoPC 16:0	460602	Phospholipid		
LysoPC 18:1	53480465	Phospholipid		
Histidine	6274	Essential amino acid		Kim et al., 2014;
Threonine	6288	Essential amino acid		Thomas et al., 2015
Valine	6287	Essential amino acid		
Methionine	6137	Essential amino acid		
Lysine	5962	Essential amino acid		
Isoleucine	6306	Essential amino acid		
Leucine	6106	Essential amino acid		
Phenylalanine	6140	Essential amino acid	✓	

L-Aspartate	5460294	Non-essential amino acid		Kim et al., 2014;
Serine	5951	Non-essential amino acid		Valarmathi et al., 2014;
Glutamine	5961	Non-essential amino acid		Thomas et al., 2015
Glycine	750	Non-essential amino acid		
Arginine	6322	Non-essential amino acid		
Alanine	602	Non-essential amino acid		
Proline	614	Non-essential amino acid		
Tyrosine	6057	Non-essential amino acid		
α-Aminobutyric acid (AABA)	6657	Non-essential amino acid		
Potassium (K)	5462222	Mineral		Valarmathi et al., 2014;
Calcium (Ca)	5460341	Mineral		Thomas et al., 2015;
Magnesium (Mg)	5462224	Mineral		Raghuvanshi et al., 2017;
Sodium (Na)	5360545	Mineral		Shozib et al., 2017;
Chromium (Cr)	23976	Mineral		Hurtada et al., 2018
Iron (Fe)	23925	Mineral		
Manganese (Mn)	23930	Mineral		
Zinc (Zn)	23994	Mineral		
Copper (Cu)	23978	Mineral		
Phosphorus (P)	5462309	Mineral		

Caproic acid	8892	Fatty acid		Valarmathi et al., 2014;
Caprylic acid	379	Fatty acid		Thomas et al., 2015
Capric acid	2969	Fatty acid		
Lauric acid	3893	Fatty acid		
Tridecanoic acid	12530	Fatty acid		
Myristic acid	11005	Fatty acid		
Pentadecanoic acid	13849	Fatty acid		
Palmitic acid	985	Fatty acid		
Stearic acid	5281	Fatty acid		
Arachidic acid	10467	Fatty acid		
9-Octadecanoic acid	965	Fatty acid		
Undecanoic acid	8180	Fatty acid		
Oleanolic acid	10494			

Myristoleic acid	5281119	Mono-unsaturated fatty acid		Valarmathi et al., 2014;
*cis*-10-Pentadecenoic acid	5312411	Mono-unsaturated fatty acid		Thomas et al., 2015
Oleic acid	445639	Mono-unsaturated fatty acid		
*cis*-Vaccenic acid	5282761	Mono-unsaturated fatty acid		
Erucic acid	5281116	Mono-unsaturated fatty acid		

Hexadecadienoic acid		Polyunsaturated fatty acid		Valarmathi et al., 2014;
Hexadecatrienoic acid	6506600	Polyunsaturated fatty acid		Thomas et al., 2015
Linoleic acid	5280450	Polyunsaturated fatty acid		
Octadecatetraenoic acid	11778225	Polyunsaturated fatty acid		
*cis*-11,14,17-Eicosatrienoic acid	5312529	Polyunsaturated fatty acid		
*cis*-5,8,11,14-Eicosatetraenoate acid		Polyunsaturated fatty acid		
Eicosatetraenoic acid	21863049	Polyunsaturated fatty acid		
Pinellic acid	9858729	Oxylipin		Kim et al., 2014
Succinic acid	1110	Carboxylic acid		
Maleic acid	444266	Carboxylic acid		
Malonic acid	867	Carboxylic acid		
Citric acid	311	Carboxylic acid		
Cinnamic acid	444539	Carboxylic acid	✓	
D-Xylose	135191	Sugar		Kim et al., 2014;
D-Fructose	2723872	Sugar		Valarmathi et al., 2014
D-Glucose	5793	Sugar		
Maltose	439341	Sugar		
*myo*-Inositol	892	Sugar		

**FIGURE 2:**
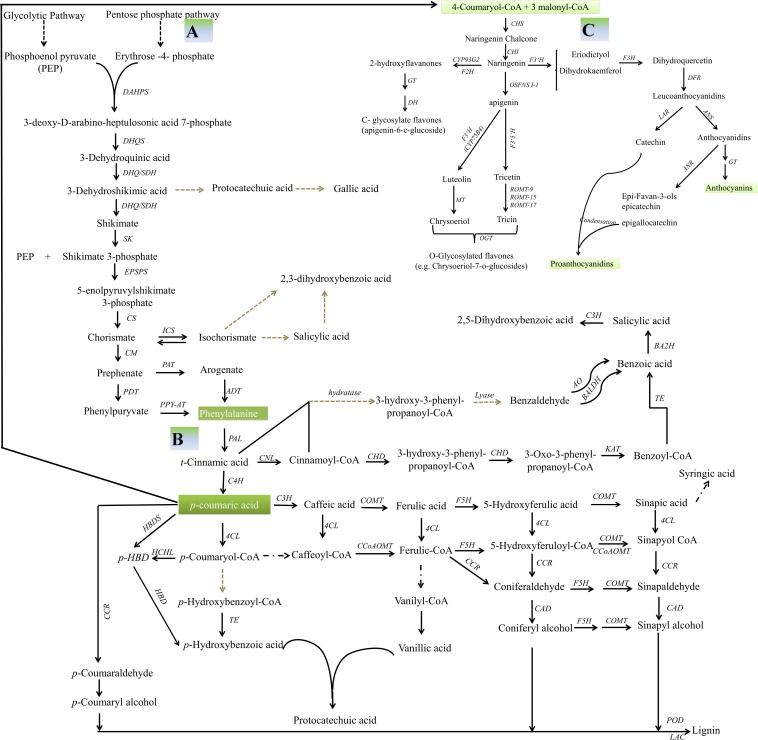
Secondary metabolism in rice. **(A)** A schematic representation of the shikimic acid pathway. DAHP, 3-deoxy-D-arabino-heptulosonic acid 7-phosphate; DAHPS, 3-deoxy-D-arabino-heptulosonate-7-phosphate synthase; DHQ/SDH, 3-dehydroquinate dehydratase/shikimate 5 dehydrogenase; DHQS, 3-dehydroquinate synthetase; DHS, 3-dehydroshikimic acid; SDH, shikimate dehydrogenase; SK, shikimate kinase; S3P, shikimic acid 3-phosphate; EPSPS, 5-enolpyruvylshikimate 3-phosphate synthase; EPSP, 5-enolpyruvylshikimate 3-phosphate; CS, chorismate synthase; CM, chorismate mutase; PAT, prephenate aminotransferase; ADT, arogenate dehydratase; PDT, prephenate dehydratase; PPY-AT, phenylpyruvate aminotransferase (Tzin and Galili, 2010; Widhalm and Dudareva, 2015; Santos-Sánchez et al., 2019). **(B)** Possible routes to the production of benzoic acid, benzoic acid-derived compounds and lignin. CNL, cinnamate-CoA ligase; CHD, cinnamoyl-CoA-dehydrogenase/hydratase; KAT1, 3-ketoacyl-CoA thiolase; TE, CoA thioesterase; BA2H, benzoic acid 2-hydroxylase; BALDH, benzaldehyde dehydrogenase; AO, aldehyde oxidase; C4H, cinnamate 4-hydroxylase; 4CL, 4-coumarate:CoA ligase; ICS, isochorismate synthase; CCR, cinnamoyl-CoA reductase; CCoAOMT, caffeoyl-CoA *O*-methyltransferase; F5H, ferulate 5-hydroxylase; CSE, caffeoyl shikimate esterase; COMT, caffeic acid *O*-methyltransferase; CAD, cinnamyl alcohol dehydrogenase; LAC, laccase; POD, peroxidase; *p*-HBD, *p*-hydroxybenzaldehyde; HBDS, 4-hydroxybenzaldehyde synthase; HCHL, 4-hydroxycinnamoyl-CoA hydratase/lyase; HBD, 4-hydroxybenzaldehyde dehydrogenase (Qualley et al., 2012; Gallage and Møller, 2015; Widhalm and Dudareva, 2015; Liu et al., 2018). **(C)** Flavonoid metabolism. PAL, phenylalanine ammonia lyase; C4H, cinnamate 4-hydroxylase; CHS, chalcone synthetase; CHI, chalcone isomerase; F3′H, flavone 3-hydroxylase; DFR, dihydroflavonol 4-reductase; ANS, anthocyanin synthase; ANR, anthocyanin reductase; GT, glucosyltransferase; LAR, leucoanthocyanidin reductase; MT, *O*-methyltransferase; F2H, flavanone 2-hydroxylase (Chen et al., 2013; Galland et al., 2014). The square dot arrows indicates steps which have not yet been fully elucidated, while the black arrows indicate steps supported by genetic evidence.

*^*a*^Compounds present in Figure 2.*

#### Carotenoids

Carotenoids represent another class of nutritionally beneficial compounds (Roberts and Dennison, 2015). Lutein and zeaxanthin represent together >90% of the carotenoids produced by rice, with carotenes, lycopenes, and β-carotene present in trace amounts (Pereira-Caro et al., 2013; Melini and Acquistucci, 2017). Most of this class of compound is present in the bran, with little or no carotenoids being found in milled rice (Petroni et al., 2017). Grain carotenoid content is a genetically variable trait, and is strongly correlated with grain pigmentation (Ashraf et al., 2017). Red and black rice accumulate a particularly high carotenoid content, while white rice accumulates very little (Ashraf et al., 2017; Petroni et al., 2017).

#### Vitamins

Rice grain represents a good source of vitamin E, including both the tocopherols and the tocotrienols (Zubair et al., 2012). The β- and γ-tocotrienols are the most abundant forms present in rice (Irakli et al., 2016). According to Gunaratne et al. (2013), red rice grains harbor higher levels of total tocopherol and tocotrienol than do the grains of modern white rice varieties. Note, however, that dehulling and milling strongly reduce the tocopherol content of the grain (Zubair et al., 2012).

#### Micronutrients

Rice grain contains traces of a number of essential micronutrients, namely zinc, magnesium, iron, copper, potassium, manganese, and calcium (Table 1; Raghuvanshi et al., 2017; Shozib et al., 2017; Shao et al., 2018). Some genetic variation in mineral content has been reported; but in general, pigmented rice grain accumulates higher amounts than does white grain rice (Shozib et al., 2017). Other studies have suggested that pigmented rice contains higher levels of zinc, iron, and manganese than does white grain, but a lower level of phosphorus (Raghuvanshi et al., 2017; Hurtada et al., 2018; Shao et al., 2018). Brown rice can provide as much as 75% of the recommended daily intake of zinc, copper, and iron, but this falls to just 37% for white rice (Hashmi and Tianlin, 2016).

### Flavonoid Metabolism in Pigmented Rice Grain

The major flavonoids present in pigmented rice grain are proanthocyanidins and anthocyanins (Table 1). The synthesis of the flavonoids is initiated by the deamination of phenylalanine to form cinnamic acid, a reaction catalyzed by phenylalanine ammonia lyase. Cinnamate 4-hydroxylase catalyses the oxidation of cinnamic acid to 4-coumaric acid, which is in turn converted to 4-coumaroyl-CoA through the action of 4-coumaroyl-CoA ligase (Cheng et al., 2014). The rate limiting step is the conversion of cinnamic acid to *p*-coumaroyl-CoA, which affects the synthesis of phenolic acids, flavanones, proanthocyanidins, and anthocyanidins (Figure 2).

#### Phenolic Acids

Compared to white grain, pigmented grain contains a higher level of phenolic acids (Gunaratne et al., 2013; Zhang et al., 2015). Cinnamic acid serves as a precursor for the synthesis of various phenolic acids, including *p*-coumaric acid, ferulic acid, sinapic acid, isoferulic acid, and 2,5-dihydroxybenzoic acid (Zhang et al., 2015; Shao et al., 2018). The predominant phenolic acids present in white rice are *p*-coumaric acid and ferulic acid; these are largely utilized as building blocks for lignin synthesis (Figure 2). In an alternative pathway, particularly active in black rice, cinnamic acid is converted to vanillic acid and protocatechuic acid (Zhang et al., 2015; Shao et al., 2018). In red rice, caffeic acid has been identified as a minor phenolic acid, while this compound is not detectable in brown rice (Gunaratne et al., 2013; Zhang et al., 2015; Irakli et al., 2016; Shao et al., 2018). Additional phenolic acids identified include syringic acid in the extract of brown, red, and black rice (Ghasemzadeh et al., 2018; Shao et al., 2018), pinellic acids in red and white rice (Kim et al., 2014), hydroxybenzoic acid in black rice extracts (Tantipaiboonwong et al., 2017), and gallic acid in the extracts of the red rice mutant AM-425 (Chiou et al., 2018). Four diferulic acids (phenolic acid dehydrodimers) are present in the insoluble-bound (Table 1; Zhang et al., 2015).

#### Flavanones

The condensation and subsequent intramolecular cyclization of three molecules of malonyl CoA and one of 4-coumaroyl-CoA is then catalyzed by chalcone synthetase to produce naringenin chalcone. Naringenin chalcone is isomerized into naringenin by the action of chalcone isomerase to form the flavones (Figure 2). Small quantities of flavones and flavanol glycosides have been detected in the grain, notably luteolin-6/8-*C*-pentoside-6/8-*C*-hexoside and certain derivatives of apigenin (Table 1). In the tricin pathway, a flavone synthase II enzyme converts naringenin to apigenin, which is then converted first to luteonin by flavonoid 3′-hydroxylase, and then to tricin by *O*-methyltransferase and chrysoeriol 5′-hydroxylase (Park et al., 2016; Figure 2). Apigenin, luteolin, tricetin, tricin, quercetin, and myricetin have all been detected in extracts of red and brown rice bran (Table 1; Galland et al., 2014; Ghasemzadeh et al., 2018). The synthesis of C-glycosylated flavanones begins with the conversion of naringenin to 2-hydroxyflavanone by flavanone 2-hydroxylase, which is then C-glycosylated by C-glucosyltransferase and finally is dehydrated by an as yet unknown enzyme (Figure 2; Du et al., 2010; Galland et al., 2014; Sasaki et al., 2014; Park et al., 2016; Poulev et al., 2019). Other flavonoid-like compounds identified in rice include quercetin-3-*O*-glucoside, quercetin-3-*O*-rutinoside, methoxy-flavanol-3-*O*-glucoside, and isorhamnetin-3-*O*-glucoside (Pereira-Caro et al., 2013; Kim et al., 2014); tricin-*O*-rhamnoside-*O*-hexoside and apigenin-6-*C*-glucosyl-8-*C*-arabinoside are particularly predominant in white rice grains (Kim et al., 2014).

#### Proanthocyanidins

Proanthocyanidins are oligomers and polymers of flavan-3-ols (Gunaratne et al., 2013). Naringenin, the universal substrate for their synthesis, is 3′-hydroxylated by flavonoid 3′-hydroxylase, producing eriodictyol, which is then converted to dihydroquercetin by the action of flavone 3-hydroxylase (Figure 2). Dihydroflavonol 4-reductase catalyses the conversion of dihydroquercetin into leucoanthocyanidins. Leucocyanidin is converted into the flavan-3-ol catechin by leucoanthocyanidin reductase, while catechin monomers are polymerized by a yet unknown pathway to form proanthocyanidin (Figure 2; Zhao et al., 2010; Galland et al., 2014). Proanthocyanidins and catechins make up the bulk of the phenolic compounds found in red rice, being responsible for the red pigmentation of the pericarp (Pereira-Caro et al., 2013; Kim et al., 2014). No proanthocyanidins have been detected in white rice accessions (Gunaratne et al., 2013), while some black rice varieties have been reported to contain them (Vichit and Saewan, 2015).

#### Anthocyanidins

The oxidization of leucoanthocyanidin to form cyanidin, pelargonidin, and delphinidin is catalyzed by anthocyanin synthase (Figure 2; Cheng et al., 2014; Galland et al., 2014). Anthocyanins, which are responsible for purple to blue pigmentation, represent the bulk of the flavonoids present in black and purple rice (Pereira-Caro et al., 2013; Zhang et al., 2015). The compounds cyanidin-3-*O*-glucoside and peonidin-3-*O*-glucoside are the most prominent, but also represented are cyanidin-3,5-diglucoside, cyanidin-3-*O*-(6″-*O*-*p*-coumaroyl)glucoside, pelargonidin-3-*O*-glucoside, peonidin-3-*O*-(6″-O-*p*-coumaroyl)glucoside, and cyanidin-3-*O*-arabidoside. Red and white rice grains have been classified as lacking anthocyanin (Gunaratne et al., 2013; Xiongsiyee et al., 2018), but both Boue et al. (2016) and Ghasemzadeh et al. (2018) have been able to detect a low level in both red and brown rice accessions. Unstable anthocyanidins can be converted into the colorless flavan-3-ols epiafzelechin, epicatechin, and epigallocatechin through the action of anthocyanin reductase, and when glycosylated, a wide array of distinct molecules are generated (Ko et al., 2006; Sasaki et al., 2014; Kim et al., 2015; Figure 2). Although the major enzymes operating in the flavonoid pathway are well known and their encoding genes have been identified (Table 2), many aspects underlying the synthesis of these pigments in rice have yet to be fully elucidated.

**TABLE 2:** Regulatory and structural genes shown to be involved in the biosynthesis of anthocyanin and proanthocyanidin in rice.

Locus	Allelic locus	Gene name	Locus ID		CHRX^*a*^	Pericarp	References
			Rice Annotation Project (RAP)	Rice Genome Annotation Project (MSU)			
Regulatory genes							
Kala1	*Rd*	OsDFR	Os01g0633500	LOC_Os01g44260	1	Red and black	Furukawa et al., 2006; Shih et al., 2008; Maeda et al., 2014; Sun et al., 2018
	*Pp*				1	Red and black	Caixia and Qingyao, 2007
Kala3		OsMYB3	Os03t0410000	LOC_Os03g29614	3	Black	Maeda et al., 2014
Kala4	*Pl*^*w*^	*OSB1*/*Pb*/*Ra*	Os04g0557800	LOC_Os04g47080	4		Hu et al., 1996; Sakamoto et al., 2001; Caixia and Qingyao, 2007; Sakulsingharoj et al., 2016
		*OSB2*	Os04g0557500	LOC_Os04g47059	4		Sakamoto et al., 2001; Sakulsingharoj et al., 2014; Oikawa et al., 2015
*Rc*	*Rc*-s	bHLH	Os07g0211500	LOC_Os07g11020	7	Red	Sweeney et al., 2006
	*Rc*				7	Red	Furukawa et al., 2006
	*rc*				7	White	Furukawa et al., 2006; Sweeney et al., 2006
	*Rc-g*				7	Red	Brooks et al., 2008
	*Rc*^*r*^				7	Red	Ferrari et al., 2015
	*Rc-gl*				7	White	Gross et al., 2010
Chromogen		*OsC1*	Os06g0205100	LOC_Os06g10350	6	Black	Saitoh et al., 2004; Rachasima et al., 2017; Sun et al., 2018
Structural genes							
Chalcone synthetase (CHS)		*OsCHS1*	Os11g0530600	LOC_Os11g32650	11	Common intermediate	Shih et al., 2008
		*OsCHS2*	Os07g0214900	LOC_Os07g11440	7	Common intermediate	
Chalcone isomerase (CHI)		*OsCHI*	Os03g0819600	LOC_Os03g60509	3	Common intermediate	Shih et al., 2008
Flavanone 3-hydroxylase (F3H)		*OsF3H-1*	Os04g0662600	LOC_Os04g56700	4	Common intermediate	Kim et al., 2008
		*OsF3H-2*	Os10g0536400	LOC_Os10g39140	10		
		*OsF3H-3*	Os04g0667200	LOC_Os04g57160	4		
Flavanone 3′-hydroxylase (F3′H)		*OsF3′H*	Os10g0320100	LOC_Os10g17260	10	Common intermediate	Shih et al., 2008
Leucoanthocyanidin reductase (LAR)		*OsLAR*	Os03g0259400	LOC_Os03g15360	3	Black rice	Kim et al., 2015
Anthocyanidin synthase (ANS)		*OsANS1*	Os01g0372500	LOC_Os01g27490	1	Black rice	Shih et al., 2008
		*OsANS2*	Os06g0626700	LOC_Os06g42130	6		
UDP-glycosyltransferase (UF3GT)		OsUGT	Os06g0192100	LOC_Os06g09240	6	Black rice	Yoshimura et al., 2012
			Os07g0148200	LOC_Os07g05420	7	Black rice	
Anthocyanin reductase (ANR)		*OsANR*	Os04g0630800	LOC_Os04g53850	4	Black rice	Kim et al., 2015

*^*a*^CHRX: chromosome.*

## The Genetic Basis of Rice Grain Pigmentation

The rice genome harbors at least two genes encoding chalcone synthetase: *CHS1* on chromosome 11 and *CHS2* on chromosome 7 (Shih et al., 2008; Han et al., 2009; Cheng et al., 2014), each contributing to flavanone biosynthesis. For the production of proanthocyanidins, three flavone 3-hydroxylase are relevant: namely *F3H-1* (chromosome 4), *F3H-2* (chromosome 10), and *F3H-3* (chromosome 4; Kim et al., 2008; Park et al., 2016). Two anthocyanin synthases are critical for the synthesis of anthocyanins, namely *ANS1* (chromosome 1) and *ANS2* (chromosome 6) (Shih et al., 2008; Table 2).

### *Rc* Role in Red Pericarp in Ancestral Rice

White grained rice was selected during rice’s domestication. The two complementary genes *Rc* (on chromosome 7), which encodes a basic helix-loop-helix (bHLH) transcription factor, and *Rd* (chromosome 1) encoding a form of dihydroflavonol 4-reductase, an enzyme which enhances the accumulation of proanthocyanidin, are together responsible for the red pericarp color. *Rc* is closely associated with shattering and grain dormancy (Sweeney et al., 2006), so therefore was selected against during domestication. *Rc-Rd* genotypes produce red grain, while *Rc-rd* genotypes produce brown grain (Furukawa et al., 2006). The three common *Rc* alleles are the wild type *Rc*, and mutant alleles *Rc-s* and *rc*. *Rc-s* differs from *Rc* due to the presence of a premature stop codon, while *rc* lacks a 14 bp stretch of the wild type sequence (Furukawa et al., 2006; Sweeney et al., 2006). Carriers of *rc* produce a colorless pericarp, while those of *Rc-s* produce a range of pericarp pigmentation (Sweeney et al., 2007). A number of variants have been identified as restoring the wild type (red) pericarp pigmentation: *Rc-g* carries a 1 bp deletion 20 bp upstream of the 14 bp *rc* deletion (Brooks et al., 2008), while *Rc*^*r*^ features a 44 bp deletion upstream of the 14 bp segment, which restores the wild type reading frame (Ferrari et al., 2015). Most varieties of African domesticated rice (*Oryza glaberrima*) produce a red pericarp, and white variants harbor a loss-of-function mutation in *Rc*. An exception is the *O. glaberrima* specific mutation *rc-gl*, which carries a premature stop codon 146 bp upstream of the site of the *Rc-s* point mutation (Gross et al., 2010).

### Regulatory Cascade Influencing Purple Rice Color

Anthocyanins are responsible for the black-purple pigmentation in rice grain. The variation seen in pigmentation intensity has been taken to imply that the trait is under polygenic control, involving as yet unidentified genes (Ham et al., 2015). A number of publications report the identification of rice genes that regulate anthocyanin production, each adopting a different gene coding system, which only adds to the confusion. According to Hu et al. (1996), two classes of regulatory gene (*R/B* and *C1/Pl*) govern both the accumulation of anthocyanin and the regulation of its deposition. Two *R* genes have been characterized: *Ra* maps to chromosome 4 and *Rb* to chromosome 1. The former gene is thought to be a homolog of the maize *R/B* gene. Three alleles of *Pl* (chromosome 4) have been identified, namely *Pl*^*w*^, *Pl*^*i*^, and *Pl*^*j*^, and each is responsible for a distinctive pattern of pigmentation. *Pl*^*w*^ activates anthocyanin synthesis in most of the aerial parts of the rice plant (although not in either the stem or the internode). The *Pl* locus harbors the two genes, *OSB1* and *OSB2*, each of which encodes a bHLH transcription factor (Table 2; Sakamoto et al., 2001). Other studies found the purple pericarp trait to be genetically determined by the dominant complementary genes *Pb* (synonym *Prp-b*) and *Pp* (synonym *Prp-a*), mapping to chromosomes 4 and 1, respectively (Table 2; Rahman et al., 2013; Ham et al., 2015). While the product of *Pb* appears to be responsible for the accumulation of pigment in the pericarp of brown grain, that of *Pp* increases the amount of the pigment, giving rise to purple grain. The number of copies of the *Pp* gene present is correlated with the intensity of the purple pigmentation (Rahman et al., 2013). In the absence of *Pp*, plants harboring *Pb* produce grain with a brown pericarp, while the pericarp of *Pp* carriers, lacking *Pb*, are white (Rahman et al., 2013). The *Pb* locus comprises of two genes, a myc transcription factor (*Ra*), along with *bHLH16*. The *bHLH16* has been shown to be involved in proanthocyanidin synthesis, while *Ra* is involved in anthocyanin synthesis. *Ra* and *OSB1* are believed to have synonymous functions (Hu et al., 1996; Sakamoto et al., 2001; Caixia and Qingyao, 2007). A 2 bp (GT) insertion in exon 7 of *Ra* abolishes purple pigmentation (Caixia and Qingyao, 2007; Lim and Ha, 2013; Rahman et al., 2013). Similarly, Sakulsingharoj et al. (2016) have found that a 2 bp (GT) insertion in exon 7 of *OSB1*, which along with a 1 bp deletion of a guanine nucleotide in exon 8, results in a threonine for methionine substitution at position 64, resulting in a white grain phenotype. Carriers of the three loci *Kala1* (chromosome 1), *Kala3* (chromosome 3), and *Kala4* (chromosome 4) express a black pericarp trait (Maeda et al., 2014). It has been suggested that *Kala4* is synonymous with *Pb*, and *Kala1* with *Pp*. *Kala4* encodes a bHLH transcription factor and corresponds to *OSB2* (Table 2). OSB2 regulates a number of genes encoding enzymes involved in anthocyanin synthesis, including *F3H*, *DFR*, and *ANS* (Sakulsingharoj et al., 2014). The chromosomal region harboring *Kala1* includes *Rd* (dihydroflavonol 4-reductase). *Kala3* is likely be a synonym of *MYB3* (Maeda et al., 2014). The black grain phenotype occurring in tropical *japonica* germplasm has been attributed to structural variants in the *Kala4* promoter sequence. Oikawa et al. (2015) have proposed that *Kala4* has been introgressed several times from *japonica* to *indica* germplasm.

The R2R3-MYB transcription factor *Os06g0205100* has been proposed as a candidate for the *C* gene, functioning as a possible activator of *DFR* and *ANS* (Saitoh et al., 2004; Rachasima et al., 2017; Sun et al., 2018). *Os01g0633500* (*A1*) is a dihydroflavonol reductase gene involved in anthocyanin synthesis (Table 2). Thus, *A1* and *C1* determine the purple color of grain. The *S1* gene (*Os04g0557500*) encodes a bHLH protein, and contributes to hull-specific pigmentation. The presence of a functional copy of both *C1* and *S1* has been shown to be required for hull pigmentation, while the product of *A1* acts as a catalyst for the development of purple hulls (Sun et al., 2018). The pattern of anthocyanin pigmentation is determined by the allelic status of *A1*, *C1*, and *S1* (Sun et al., 2018). Several authors have attempted to correlate sequence variants of a number of regulatory genes, e.g., *C1* and *OSB2*, with phenotypic variation in rice grain pigmentation (Sakulsingharoj et al., 2016; Rachasima et al., 2017; Sun et al., 2018). Lachagari et al. (2019) conducted comparative genomics in 108 rice lines and identified novel allelic variants in a number of genes belonging to the flavonoid pathway, cytokinins glucoside, and betanidin degradation biosynthesis that were associated with purple pigmentation. Although a number of genes responsible for grain pigmentation have already been identified (Table 2), there is still a possibility that additional genes and variants thereof, remain to be discovered.

### The Genetic Basis of Grain Pigmentation Inferred From Quantitative Trait Loci Analysis or Genome Wide Association Studies

A number of attempts have been made to exploit the quantitative trait loci (QTL) mapping approach as a means of inferring the genetic basis of grain pigmentation (Table 3). Tan et al. (2001) identified nine QTL in an analysis of flour pigmentation in a recombinant inbred line (RIL) population. Three QTL reflected variation in the CIE 1976 color parameter L^∗^ (lightness), two in a^∗^ (red-green), and four in b^∗^ (yellow-blue). In a backcross RIL population, made from a cross between the rice varieties “Kasalath” (red pericarp) and “Koshihikari” (white pericarp), Dong et al. (2008) identified four QTL underlying variation in red pigmentation, with the two largest effect QTL co-locating with *Rc* and *Rd*, the two minor effect QTL being novel. An analysis carried out by Matsuda et al. (2012) suggested that flavonoid content was governed by genetic factors which control flavone glycosylation. In a recent study 21 QTL, responsible for variation in the content and composition of anthocyanin and proanthocyanidin, were identified (Xu et al., 2017). While some mapped to locations occupied by already known genes, others mapped to genomic regions not previously identified as harboring genes involved in rice grain pigmentation.

**TABLE 3:** Quantitative trait loci identified for colored related traits, anthocyanin and proanthocyanidin.

No.	Population	Size	Markers	Trait category	QTLs/QTNs	Closest structural and/or regulatory genes	Chromosome	Effect (%)	References
1	RILs	238	162 RFLP and 48 SSRs	Flour color	9		1,3,4,5,6,7,8	4.3–25.4	Tan et al., 2001
					L* (3)		5,6,8	4.5–15.7	
					a* (2)		4,7	6.9–10.5	
					b* (4)		1, 3,6,8	4.3–25.4	
2	BRILs	182	162 RFLP	Degree of red coloration	4		1,7,9,11	2.1–83.7	Dong et al., 2008
					*q*DRC-1*	*Rd*	1	3.6–3.7	
					*q*DRC-7*	*Rc*	7	75.9 -83.7	
					*q*DRC-9		9	2.1–3.2	
					*q*DRC-11		11	3.3–3.4	
3	RILs	182	126 SSRs	Anthocyanin and proanthocyanidin	21		1,2,3,7,8,10,12	3.8–34.8	Xu et al., 2017
					ANC (8)		1,2,3,7,10	8.8–34.8	
					PAC (13)		1,2,7,8,10,12	3.8–17.0	
4	Diversity panel	416	100 SSRs and 10 gene markers	Grain color	25		1,4,6,7,8,9,10,11,12	1.39–86.68	Shao et al., 2011
					L* (3)	*Ra*, *Rc*	4,7,10	4.96–31.23	
					a* (8)	*Ra*, *Rc*	1,4,7,8,9,11,12	1.51–19.65	
					b* (6)	*Ra*	4,6,8,9,10	4.38–49.82	
					c (3)	*Ra*	4,8,10	1.39–3.99	
					H° (5)	*Ra*	4,6,8,9	5.4–86.68	
				Phenolic and flavonoid content	10		4,7,8,9,10	2.64–39.67	
					PC (4)	*Ra*, *Rc*	4,7,8,9	5.87–39.67	
					FC (6)	*Ra*, *Rc*	4,7,8,9,10	2.64–35.35	
5	Diversity panel	203	sequencing data	Pericarp color (PC)	4		7,10		Wang et al., 2016
					*Rc-s*	Rc-s	7		
					*qPc10*	F3H	10		
6	Diversity panel	244	122,785 SNPs	Red seed color	snp_07_6067391	bHLH			Butardo et al., 2017
7	Diversity panel	419	208,993 SNPs	Pericarp color_whole panel	763		1,3,4,7,8,10,11		Yang et al., 2018
						*Rd*	1		
						MYB family transcription factors	10,11		
						WD domain, G-beta repeat domain containing protein	8		
				Pericarp color_*indica*	99				
						MYB family transcription factors	10,11		
						*OsCHI*	3		
						Kala4	4		
						*Rc*	7		
						WD domain, G-beta repeat domain containing protein	8		
						*OsCHI*	3		
						Kala4	4		
						*Rc*	7		

*RILs, recombination inbred lines; BRILs, backcross-recombinant inbred lines.*

The genome wide association study (GWAS) approach, which has certain advantages over QTL mapping (Korte and Farlow, 2013), has been applied in a few cases to determine the genetic basis of grain pigmentation (Table 3). Shao et al. (2011) used GWAS to identify 25 marker–trait associations for grain pigmentation: some related to pigment intensity, others to hue angle, L^∗^, a^∗^, or b^∗^. Their analysis confirmed the importance of *Ra* and *Rc*. Butardo et al. (2017) used GWAS to uncover a number of single nucleotide polymorphism loci (SNPs) linked to *Rc*. The 763 SNPs associated with pericarp pigmentation uncovered by Yang et al. (2018) mapped to 6 of the 12 rice chromosomes (chromosomes 1, 3, 4, 8, 10, and 11); some of the most significantly associated SNPs lying close to previously identified structural or regulatory genes, but others map to regions not previously associated with variation in rice grain pigmentation.

### ‘Omics Approaches Taken to Unraveling the Mechanistic Basis of Grain Pigmentation

High throughput genomics, including transcriptomics, proteomics, and metabolomics, have contributed to the unraveling of biochemical pathways underlying target traits. By combining genetics with systems biology tools the target genomic regions were narrowed down to identify candidate genes and proteins influencing key nutritional traits of interest in rice (Butardo et al., 2017; Anacleto et al., 2019).

Differential transcriptomic analyses between pigmented and non-pigmented rice grains identified regulators and downstream targets of flavonoid pathway genes (Oh et al., 2018). The high anthocyanin content of black rice was associated with enhanced transcription of genes encoding anthocyanidin synthase, while high proanthocyanin content, characteristic of red rice, was accompanied by a notable abundance of transcript for a gene encoding leucoanthocyanidin reductase (Chen et al., 2013). Transcript abundance of genes encoding chalcone synthetase, chalcone isomerase, flavanone 3-hydroxylase, dihydroflavonol 4-reductase, and anthocyanin synthetase was compared in white, black, and red rice grain by Lim and Ha (2013). Four genes were markedly up-regulated in pigmented grain varieties, while the gene encoding chalcone isomerase displayed a similar level of transcription in both white and pigmented varieties. The enhanced abundance of transcripts of chalcone synthetase, flavanone 3-hydroxylase, and anthocyanin synthetase seen in some black varieties implied a strong correlation between transcription and pigment content. Sun et al. (2018) found that flavonoid pathway genes were regulated by ternary MYB-bHLH-WD40 transcriptional complexes (Xu et al., 2013; Zhang et al., 2018). A microarray-based comparison of black and white rice identified nearly 1,300 differentially transcribed genes, of which 137 were predicted to encode transcription factors belonging to 1 of 10 different classes (Kim et al., 2011). When Kim et al. (2018) applied RNA-seq to analyze differential transcription, it was concluded that the B-box protein encoded by *BBX14* was a key regulator of the anthocyanin synthesis pathway. Anthocyanin production in pigmented grain appeared to be induced and fine-tuned by BBX14 in conjunction with the basic leucine zipper transcription factor HY5. Both irradiation at a high light intensity and the plant’s sugar content can influence anthocyanin and proanthocyanin synthesis (Xu et al., 2013; Ma et al., 2018; Zhang et al., 2018). Therefore it would be of value to search for linkages between photoreceptor and light signal transduction elements associated with anthocyanin/proanthocyanidin synthesis in pigmented rice (Teng et al., 2005). While transcriptomic analyses have succeeded in shedding some light on the transcriptional regulation of secondary metabolites, unraveling post-transcriptional and post-translation processes may well provide further insights into the identity of the rate-limiting steps of grain pigmentation (Merchante et al., 2017; Spoel, 2018).

The abundance of a given transcript and its translation product are not always linearly related, due to post-transcriptional regulation, translation and post-translational processing, and peptide modification (Chen et al., 2017; Zhang et al., 2017). Thus, a proteomic analysis can give a more nuanced picture of the differences between pigmented and non-pigmented grains, than is possible from a transcriptomic analysis. A total of 230 differentially abundant proteins, involved in various metabolic processes, were identified by Chen et al. (2016) from a comparison between black and white grains sampled at five stages of grain development. A number of proteins involved in the synthesis of flavonoids and sugars were found to be more abundant in the black grain, while proteins associated with signal transduction, redox homeostasis, photosynthesis, nitrogen metabolism, and tocopherol synthesis were less abundant. In particular, chalcone synthetase was pinpointed as a key component required for the synthesis of anthocyanin.

Metabolomic analyses have also been successfully used to characterize the cellular composition of rice (Table 1). Comparative metabolome studies of black, red, and non-colored rice revealed that various anthocyanins, tocopherol, fatty acid methyl esters, free sugars, and fatty acids were found to be significantly different (Frank et al., 2012). de Guzman et al. (2017) were able to monitor more than 1,000 metabolites in a screen of several rice varieties differing with respect to their nutritional quality and glycemic response (de Guzman et al., 2017). Comparisons of the grain metabolomes of diverse rice accessions have revealed that a substantial degree of variation exists at this level (Gong et al., 2013; Pereira-Caro et al., 2013). However, the grain metabolome is highly dynamic, responding strongly to the plant’s external environment, so this variation is as a consequence of genetic and environmental variation and the interaction there-of. Correlation analyses carried out between individual metabolites have nevertheless revealed the regulation of the grain metabolome, with clusters of co-accumulated metabolites appearing to be under the control of shared genetic factors (Matsuda et al., 2012, 2015).

The application of ‘omics-based platforms have begun to reveal the genetic and biochemical basis of the grain pigment parameters a^∗^, b^∗^, L^∗^, hue, and chroma. While transcriptomic and metabolomic analyses have identified certain important structural and regulatory genes influencing core components of flavonoid synthesis (Lee et al., 2015; Oh et al., 2018), what is still lacking is a comprehensive understanding of the molecular machinery underlying key metabolic processes such as the polymerization and transport of tannins. A more integrated approach, focusing on identifying linkages between regulatory networks is needed (Figure 2). Combining diverse datasets facilitates the reconstruction of regulatory networks and the identification of key modulators (Butardo et al., 2017; Wambugu et al., 2018). As an example, an exploration of the key genetic influences affecting grain amylose–amylopectin composition has implicated two genomic regions, one on chromosome 6 and the other on chromosome 7 (Butardo et al., 2017). The genetic region on chromosome 7 is in the vicinity of *Rc* and includes a haplotype associated with increased amylose and reduced accumulation of short chain amylopectin. The bHLH transcription factor encoded by *Rc* activates the gene encoding dihydroflavonol 4-reductase, thereby influencing the formation of red pigmentation. The same transcription factor has also been proposed to act as a regulator of starch structure, as it engages within the network regulating granule-bound starch synthase activity.

### Conserving and Utilizing Pigmented Rice Landraces for Future Breeding

Although rice landraces with pigmented grain represent an important genetic reservoir for rice improvement, these populations are rapidly being lost as a result of the introduction of more productive, modern white rice varieties. Some of these materials have been safeguarded in *ex situ* gene banks, such as the major gene bank curated by the International Rice Research Institute^2^. Few of these landraces have been systematically characterized in terms of their grain end-use quality, their nutritional features and potential health benefits. Therefore, there is an urgent need to validate the traditional knowledge associated with these materials with scientific-based analyses. While the productivity of the landrace materials is undoubtedly lower than that of modern white rice varieties, their market value is potentially quite high, given the growing consumer preference for nutritious foods (Islam et al., 2018). Looking forward, there is a major opportunity for breeding programs to develop productive pigmented varieties (Voss-Fels et al., 2019).

Understanding the mode of inheritance of grain pigmentation, identifying beneficial alleles of the key genes underlying these traits, and developing trait-specific markers, will contribute to accelerating efforts to breed high yielding pigmented rice varieties. Advanced generation breeding lines of pigmented lines have been developed (Bhuiyan et al., 2011; Arbelaez et al., 2015). A black rice line has been developed in the genetic background of a leading Japanese white rice variety (Koshihikari); which has eating quality superior to that of the widely cultivated black rice variety “Okunomurasaki” (Maeda et al., 2014). Crosses have been initiated between pigmented and non-pigmented varieties to develop pigmented varieties adapted to the growing conditions in Kazakhstan (Rysbekova et al., 2017). The Thai aromatic, deep purple *indica*-type rice variety “Riceberry” has developed a reputation for its health-promoting properties. Riceberry combines the desirable features of two prominent rice varieties, one a local, non-glutinous purple rice and the other an aromatic white jasmine rice (Waiyawuththanapoom et al., 2015; Gene Discovery Rice and Rice Science Center, 2017). Two improved pigmented varieties (the red rice “Rubi” and the black rice “Onix”) have been released in Brazil (Wickert et al., 2014).

## Conclusion

Pigmented rice varieties are gaining popularity among consumers, and demand is only expected to rise. The seed supply chain of pigmented rice is weak and thus rice value chain opportunities have to evolve to meet the current nutritional demand. Production of pigmented rice using landraces is unable to meet market demand, emphasizing the need to genetically improve these landrace materials. Systematic nutritional characterization of the 130,657 accessions curated by International Rice Research Institute’s gene bank and Africa Rice^3^, which including pigmented entries, will create new avenues for nutritional diversification that reaches lower income target countries. These, as well as other, national *ex situ* collections, represent a valuable source of genetic variation for the improvement of pigmented rice, providing materials to elucidate the genetic basis of grain pigmentation and associated nutrition-related traits. The process of identifying as yet unknown genes influencing flavonoid metabolism and grain pigmentation could be accelerated by whole genome re-sequencing, allowing novel allelic variants to be harnessed for use as markers. Fine mapped genetic regions associated with proanthocyanidins and anthocyanin needs to be undertaken to develop quality markers to support marker-assisted-selection breeding of these nutritional traits into high yielding rice backgrounds. A systems approach to study implication of diet based health benefits would require holistic understanding of the molecular basis of human health benefits of consuming grain pigmentation, enabling the identification of the modulators involved to overcome the prevailing double burden malnutrition and communicable diseases in the target communities. While several health benefits were shown to possess to consume pigmented rice, its texture and palatability is found to be poor and thus its acceptance rate is lower. To address this limitation, we need to explore the genetic variation for the retention of flavonoids in the milled endosperm.

## Author Contributions

EM and NS drafted the manuscript. TK, HJ, NE, CB, and LB edited the part of the sections.

## Conflict of Interest

The authors declare that the research was conducted in the absence of any commercial or financial relationships that could be construed as a potential conflict of interest.

## Footnotes

1 https://www.un.org/development/desa/en/news/population/world-population-prospects-2019.html

2 www.irri.org/international-rice-genebank

3 www.genebanks.org/genebanks/africarice/
